# The Structure of Co-Occurring Bullying Experiences and Associations with Suicidal Behaviors in Korean Adolescents

**DOI:** 10.1371/journal.pone.0143517

**Published:** 2015-11-30

**Authors:** Beop-Rae Roh, Yoewon Yoon, Ahye Kwon, Seunga Oh, Soyoung Irene Lee, Kyunghee Ha, Yun Mi Shin, Jungeun Song, Eun Jin Park, Heejung Yoo, Hyun Ju Hong

**Affiliations:** 1 Suicide and School Mental Health Institute, Anyang, Republic of Korea; 2 University of Southern California, School of Social Work, Los Angeles, CA, United States of America; 3 Department of Psychiatry, Hallym University Sacred Heart Hospital, Anyang, Republic of Korea; 4 Seoul National Hospital, Seoul, Republic of Korea; 5 Department of Psychiatry, Soonchunhyang University Bucheon Hospital, College of Medicine, Soochunhyang University, Gyeonggi-Do, Republic of Korea; 6 School of Humanities and Social Science, Ajou University, Gyeonggi-do, Republic of Korea; 7 Department of Psychiatry, Ajou University School of Medicine, Suwon 443–721, Republic of Korea; 8 Department of Psychiatry, National Health Insurance Service Ilsan Hospital, Goyang, Republic of Korea; 9 Department of Psychiatry, Ilsan Paik Hospital, Inje University College of Medicine, Goyang, Korea; 10 Department of Psychiatry, Seoul National Bundang Hospital, Seongnam, Republic of Korea; University of Vienna, School of Psychology, AUSTRIA

## Abstract

**Objective:**

This study had two main goals: to examine the structure of co-occurring peer bullying experiences among adolescents in South Korea from the perspective of victims and to determine the effects of bullying on suicidal behavior, including suicidal ideation and suicide attempts, among adolescents.

**Method:**

This study used data gathered from 4,410 treatment-seeking adolescents at their initial visits to 31 local mental health centers in Gyeonggi Province, South Korea. The structure of peer bullying was examined using latent class analysis (LCA) to classify participants’ relevant experiences. Then, a binomial logistic regression adjusted by propensity scores was conducted to identify relationships between experiences of being bullied and suicidal behaviors.

**Results:**

The LCA of experiences with bullying revealed two distinct classes of bullying: physical and non-physical. Adolescents who experienced physical bullying were 3.05 times more likely to attempt suicide than those who were not bullied. Victims of (non-physical) cyber bullying were 2.94 times more likely to attempt suicide than were those who were not bullied.

**Conclusions:**

Both physical and non-physical bullying were associated with suicide attempts, with similar effect sizes. Schools and mental health professionals should be more attentive than they currently are to non-physical bullying.

## Introduction

School bullying is a worldwide problem among adolescents, affecting the academic achievement, social skills, and psychological well-being of targeted individuals[1]. Although school bullying is not a well-defined term in academic literatures, previous studies on bullying has suggested measurements, prevalence and characteristics differed by gender, age, and the nations.

Olweus [2]identified three core elements of bullying: 1) aggressive behavior that involves unwanted negative actions; 2) behaviors that are repeated over time; and 3) an imbalance of power or strength. Adolescents may be involved in bullying as victims, perpetrators, or victim-perpetrators. Bullying can be categorized with respect to various expressions of aggression as direct (overt) bullying and indirect (covert, relational) bullying. Direct bullying includes physical and verbal aggression. Indirect bullying includes aspects of relational or social isolation such as ignoring, excluding, and backbiting. Direct bullying is more frequently observed among boys, whereas indirect bullying is more frequent among girls [2–5].

Being the victim of school bullying has been associated with multiple negative mental and physical health consequences[3,4], and repeated experiences of school bullying are directly or indirectly connected to depressive tendencies and suicidal behavior[6,7]. One study found that students who have experienced school bullying are twice as likely to report suicidal ideation and 40% more likely to suffer from depressed thoughts compared with those who have not had these experiences[8]. Similarly, in research among suburban populations in the US, bullying was found to be associated with suicidal ideation and suicide attempts [9–11]. Kim and Leventhal [12]] review of 37 studies found a consistent association between being bullied and suicidal thoughts among youth. Both perpetrators and victims of bullying are at higher risk for depression, suicidal ideation, and suicide attempts than are adolescents not involved in bullying [5,13,14].

Most studies pertaining to bullying and suicide among adolescents have investigated the various effects of bullying among victims, perpetrators, and victim-perpetrators. Many studies have focused on combinations of various types of bullying using standardized questionnaires, but have not dealt with the effects of these bullying types in victim’s perspective on suicide. In the few studies that have investigated the effects of different types of bullying on suicide [5,15–17], bullying was classified differently depending on the measurement instrument. In reality, victims of bullying are more likely to experience various forms of bullying simultaneously than to experience a single form at a time. Until now, no studies have examined the patterns of co-occurring peer bullying experiences from the perspective of individual victims. Such research is important for the following two reasons. First, understanding bullying from the vantage point of individual victims will result in more realistic definitions and conceptualizations. Second, information regarding the effects of specific bullying types on mental health can lead to more appropriate interventions.

This study was designed to meet two goals. First, we sought to examine the structure of peer bullying in school environment in South Korea using a relatively large sample of adolescents who visit to local mental health center for further treatment or evaluation. To this end, we used latent class analysis (LCA) to analyze the structures of bullying from the victims’ perspective and to categorize experiences of being bullied. Second, we attempted to identify the effects of these experiences on suicidal behaviors, including suicidal ideation and suicide attempts, by controlling for potential confounding factors. We hypothesized that several distinctive bullying victimization types would be identified. For example, overt bullying is more physical, and covert bullying is more social and non-physical. We hypothesized that each type would exert a distinct effect on suicidal ideation and attempts among adolescents.

## Methods

This study was conducted through the Gyeonggi provincial mental health center at 31 local mental health centers in collaboration with the Gyeonggi Provincial Office of Education. Gyeonggi Province is 10,172.07 km^2^ in size and comprises industrial, urban, and rural areas; the province had a population of 12,789,445 in 2014. The study used data obtained during the initial assessments of adolescents who visited 31 local mental health centers in Gyeonggi province, Korea, in 2012. About three-fourths of the sample was referred by schools through the Children and Adolescents’ Mental Health Promotion Project (CAMHP); the remainder were self-referred or referred by a source in the community. The first step of CAMHP was to screen for middle and high school students at high risk for emotional and behavioral problems using a screening questionnaire. Individual assessment and treatment were subsequently provided at local mental health centers or psychiatric clinics. When individuals visited local mental health center, they were asked to complete several questionnaires and participate in an interview conducted by a mental health professional. A total of 4,410 participants provided data (age range: 12–19 years). The protocol for this study was approved by the Institutional Review Board of Hallym University Sacred Heart Hospital. Written informed consent for participation in the study was obtained from parents and the adolescents after the nature of the study was fully explained.

### Demographic Characteristics

We asked adolescents and their parents to complete questionnaires requesting demographic information including sex, age, parents’ education, family structure, and socioeconomic status according to social welfare status. Family structure was categorized as follows: two parents, single parent, and other. Social welfare status was categorized as follows: families living below the national poverty line receiving public assistance and others.

### Bullying Experiences

Participants completed the self-administered questionnaire about their experiences of being the victims of various types of bullying during the past 12 months, providing yes/no responses to the questions. Participants were asked whether they had been the targets of bullying in the form of “physical bullying (hitting), verbal bullying (threatening or intimidation), extortion (having their belongings taken), ostracism by a group, harassment (being forced to do work), sexual bullying, and cyber bullying.”

These bullying types were chosen by our research team in consideration of the Korean situation. These seven types of bullying experiences were thought to be the most frequent forms of bullying in Korean schools. Ostracism, a common form of bullying in Korea, is a kind of social bullying in which victims are excluded from all kinds of social relationships and treated as if they were invisible, typically by peer groups rather than by an individual. Participants were permitted to check multiple items if appropriate.

### Suicidal Behaviors

Suicidal ideation and behavior were assessed with self-reports. Participants who provided affirmative responses to the statement “I have said that I wanted to die during the past 3 months” were classified as having suicidal ideation. Those providing affirmative answers to “I have attempted suicide during the past 3 months” were classified as having made a suicidal attempt.

### Clinical Variables

We assessed emotional and behavioral problems using the Strengths and Difficulties Questionnaire (SDQ). The SDQ is designed to screen for mental health problems among children and adolescents and is used in both community and clinical settings throughout the world (http://www.sdqinfo.org). This tool contains 25 items, 20 of which are related to problem areas such as peer relationships, hyperactivity and inattention, conduct disorders, and emotional symptoms. The remaining five items comprise a prosocial behavior scale. We used these five clinical variables to calculate the propensity scores for suicidal behaviors. The psychometric properties of the Korean version of the SDQ (SDQ-Kr) for parents have been demonstrated to be acceptable compared with other translated versions.[18] The self-rated SDQ-Kr is currently undergoing standardization, and we used the preliminarily translated form of the self-rated SDQ-Kr. With regard to internal consistency, we calculated the Cronbach’s α of five scales: peer problems, 0.64; hyperactivity/inattention, 0.76; conduct disorder, 0.47; emotional symptoms, 0.77; and prosocial behaviors, 0.62. When comparing to western countries, the alpha for hyperactivity/inattention, emotional symptoms and prosocial behaviors subscales is similar. On the other hand, peer problems subscale is higher, and conduct problems subscale is lower [19,20]. However, all α coefficients appears to be higher than China.[21].

## Data Analysis

We used LCA to examine co-occurrences among various types of bullying experience and to understand the structure of bullying using the poLCA package in R[22]. LCA identifies classes of individuals with similar experience patterns as measured by dichotomous variables. The latent class model with the best explanatory power is selected according to the parsimony of the model. For this purpose, we determined the best-fitting model by comparing the Akaike information criterion (AIC) and the Bayesian information criterion (BIC) among models. Lower values for both criteria represent a better-fitting model, taking parsimony into account. The meaning of the latent classes is interpreted based on the estimated item-response probabilities. To identify demographic and clinical differences among these classes, we used ANOVA and a chi-squared test.

To examine the relationship between latent classes and suicidal behaviors, we calculated propensity scores weighting the binominal logistic regression using the twang package in R[22]. Propensity scores are primarily used to estimate the probability of a treatment or intervention (bullying experiences are considered treatments for the purposes of our study) for each case by accounting for the covariates that predict them. This method has advantages in controlling for confounding variables and selection bias and for comparing different treatment effects in a quasi-experiment.

Our assumption that there were more than two bullied groups (i.e., non-bullied groups and various types of bullied groups), allowed us to use multinomial logistic regression analysis to estimate multiple propensity scores. Propensity scores estimate the average treatment effect (ATE). In this study, the ATE of experiences with bullying type A compared with experiences with bullying type B represents a comparison of the mean outcomes of both A and B in the total population under examination[23]. The propensity scores for each type of bullying experience were estimated using the regression formula for the reference group, namely the non-bullied group. Sex, age, family type (two parents or other), paternal education, social welfare status, hyperactivity, emotional problems, conduct problems, peer relationship problems, and the prosocial subscale of the SDQ-Kr were treated as confounding variables in this calculation. Because it was possible that more than three groups would be compared simultaneously in this study, a boosted multinomial logistic regression analysis was used for the propensity scores. Finally, we calculated propensity scores using weighted binomial logistic regression analysis. In this stage, we examined the relationship between experiences of being bullied and suicidal behaviors, considering any compounding effects among them.

## Results

The average age of participants was 14.3 years; 43.2% of participants were male and 56.8% were female. A total of 77.1% of participants lived with two parents. With regard to suicidal behaviors, 39.6% of our participants (n = 1,746) reported that they had experienced suicidal ideation, and 6.5% of this group (n = 287) had actually attempted suicide. The relatively high proportion of those with suicidal ideation may be partially related to our inclusion of a high-risk group in our sample. A total of 851 adolescents (19.3%) responded that they had experienced at least one of the types of bullying listed on the questionnaire, while the remaining 3559 adolescents (80.7%) responded that they had not experienced any type of bullying. The most commonly experienced type of bullying was verbal bullying (10.8%), which showed a relatively high prevalence, followed by ostracism (7.9%), physical bullying (6.1%), cyber bullying (3.0%), harassment (2.8%), extortion (1.5%) and sexual bullying (0.5%).

**Fig 1:**
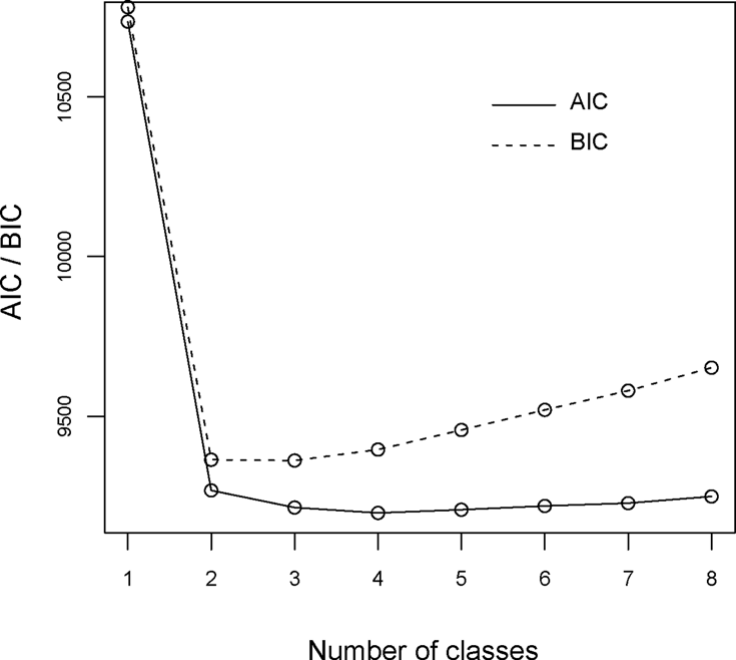
Scree plot of AIC and BIC.

Fig 1 represents a scree plot displaying AIC and BIC values for the LCA models. The scree plot shows differences in AIC and BIC values according to the number of classes of bullying experienced. The dotted line indicates changes in BIC, and the solid line indicates changes in AIC. Largest declines in the BIC or AIC is usually used as optimal model selection criteria. Two-class solution of majority of non-experienced (92.7%) and experienced (7.3%) was the largest decline in the BIC and AIC. However, we chose an alternative solution that includes more detailed bullying classes to properly classify bullied experiences. The two indicators show slightly different results. According to the BIC, the lowest value indicates that a three-class solution had the best fit and the four-class solution had even better model fit according to the AIC. Therefore, we chose the three-class solution considering the simplicity.

**Fig 2:**
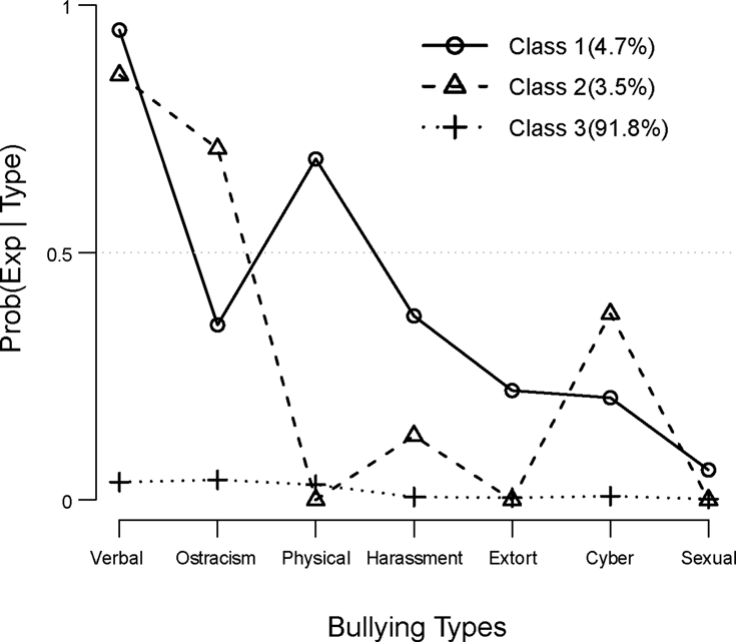
LCA classes and bullying types.

Fig 2 shows the LCA classes and the distribution of bullying experiences. A total of 4.7% of participants were categorized into Class 1, which includes primarily physical and verbal behaviors that we labeled as “physical.” Class 2, which involves verbal bullying, ostracism, and cyber bullying, but not physical bullying, was labeled as “non-physical” and included 3.5% of the total sample. Class 3, labeled “non-experienced” includes participants who had not experienced any kind of bullying. Verbal bullying was the most common experience among members of Classes 1 and 2. However, participants in Class 2 reported more covert and social features and had no experience with physical bullying.

Table 1 represents the association between bullying experiences and reconstructed bullying classes. Most notably, physical bullying was shown to be high in the physical class, at 80.2%. For both the physical and non-physical classes, verbal bullying exhibited the highest proportion among the seven types of bullying experiences, 97.3% and 93.0%, respectively. In the non-physical class, forms of ostracism and cyber bullying exhibited higher proportions (87.3% and 45.8% respectively) than in the physical class.

**Table 1:** Association between Bullying Classes and Bullying Types.

BullyingTypes	Bullying Classes			
	Non-experienced (%)(n = 4086)	Physical bullying (%)(n = 182)	Non-physical bullying (%)(n = 142)	χ^2^
**Verbal**	4.1	97.3	93.0	2599.43***
**Ostracism**	4.1	31.3	87.3	1456.32***
**Physical**	3.0	80.2	0.0	1822.99***
**Harassment**	0.5	43.3	14.8	1269.20***
**Extort**	0.5	25.3	0.0	740.72***
**Cyber**	0.8	19.8	45.8	1141.98***
**Sexual**	0.2	6.6	0.0	150.03***

*** p < .001

The clinical and demographic characteristics of each bullying class are shown in Table 2. Males who had experienced being bullied were most frequently categorized into the physical class (68.1%), whereas bullied females were most frequently categorized into the non-physical class (80.3%). Additionally, both of the groups that had experienced bullying were significantly different from the non-experienced group with respect to the prevalence of economic hardship (10.4% in the non-physical bullying class and 13.4% in the non-physical bullying class, compared with 7.2% in the non-experienced class). From a clinical point of view, scores on all four clinical subscales of the SDQ-Kr except for the prosocial behavior subscale (i.e., hyperactivity, emotional problems, conduct problems, peer problems) were significantly higher for the groups that had experienced bullying compared with the non-experienced group; no significant clinical differences were found between the physical and non-physical classes.

**Table 2:** Clinical and Demographical Characteristics of Victim of Bullying.

Characteristics	Bullying Classes			
	Non-Experienced(n = 4086)	Physical bullying(n = 182)	Non-physical bullying(n = 142)	χ^2^, F
**Sex, %**				
**Male / Female**	42.9/57.1	68.1/31.9	19.7/80.3	χ^2^ = 78.1***
**Age(year), mean(SD)**	14.4(2.6)	14.2(3.7)	13.8(1.1)	F = 4.0*
**Family Type, %**				
**Two parents / Other Type**	77.3/22.7	70.8/29.2	78.9/21.1	χ^2^ = 5.3
**Father’s Education(year), mean(SD)**	12.4(3.4)	12.0(3.7)	12.6(3.7)	F = 1.7
**Economic Hardship, %**				
**Poverty / Non-poverty**	7.2/92.8	10.4/89.6	13.4/86.6	χ^2^ = 13.4*
**SDQ(score)**				
**Hyperactivity, mean(SD)**	4.8(2.4)	6.0(2.3)	5.7(2.3)	F = 28.3*** (P = NP>NE)
**Emotional problem, mean(SD)**	4.3(2.7)	6.1(2.5)	6.4(2.4)	F = 81.3*** (P = NP>NE)
**Conduct problem, mean(SD)**	3.4(1.7)	4.4(1.9)	3.9(1.8)	F = 32.3*** (P = NP>NE)
**Peer problem, mean(SD)**	2.1(1.7)	4.5(2.5)	4.2(2.5)	F = 248.6*** (P = NP>NE)
**Prosocial behavior, mean(SD)**	5.7(1.9)	5.5(2.0)	5.3(2.2)	F = 2.9

Note: Tukey’s honest significant difference test is conducted for group comparisons. NE; non-experienced, P; Physical bullying, NP; non-physical bullying.

*p < .05

***p < .001

Table 3 shows the results of the binary logistic regression analysis examining the relationship between bullying class and two suicidal behaviors. The non-adjusted regression analysis without propensity score-weighted suicidal ideation showed that both the physical class (OR = 3.43, p < .01) and the non-physical class (OR = 2.24, p < .01) were more likely to report suicidal ideation compared with the non-experienced group. However, the regression coefficients of the adjusted analysis weighted for propensity scores were lower and not statistically significant. This result indicates that the association between peer bullying and suicidal ideation was weak and that various confounding variables may have been involved.

**Table 3:** Adjusted Associations between Bullying Classes and Suicidal Behaviors.

Bullying Classes	Suicidal Ideation		Suicidal Attempt	
	Regression coefficient(OR)	Adj. regression coefficient(OR)	Regression coefficient(OR)	Adj. regression coefficient(OR)
**Non-experienced (reference)**	(1.00)	(1.00)	(1.00)	(1.00)
**Physical bullying**	1.233**(3.43)	.535(1.71)	1.167***(3.21)	1.118*(3.06)
**Non-physical bullying**	.807**(2.24)	.400(1.49)	1.187***(3.28)	1.079**(2.94)

Note: OR means odds ratio in this table. Adjusted regression coefficients were estimated after propensity scores weighting procedures. Sex, age, family type (two parents or other), paternal education level, economic hardship, hyperactivity, emotional problems, conduct problems, peer relationship problems, and the prosocial subscale of the SDQ-Kr were confounding variables in calculation of propensity scores.

*p < .05

**p < .01

***p < .001

In terms of suicide attempts, the regression coefficients between bullying experience and suicide attempts for both the physical and non-physical classes were statistically significant in the non-adjusted model. Both the physical class (OR = 3.21, p < .001) and the non-physical class (OR = 3.28, p < .328) were more likely to attempt suicide than was Class 3. Even after weighting by propensity scores, these associations remained statistically significant. Both the physical class (OR = 3.06, p < .05) and the non-physical class (OR = 2.94, p < .01) were more likely than the reference group to attempt suicide after adjustment. This result reflects the strong association of both classes of victimization with suicide attempts even after controlling for confounding variables.

## Discussion

This study identifies patterns of co-occurring peer bullying experiences from the perspective of victims, and the association of these experiences on suicide in a relatively large sample of treatment-seeking adolescents. Experiences of being bullied were classified into two distinct classes. The most noticeable difference between the physical class and the other classes involved the presence or absence of a physical bullying experience. The non-physical class included more of the social aspects of experiences with bullying, but both classes were associated with suicide attempts, with similar effect sizes. These patterns were kept after controlling compounding factors using propensity scores.

This study found that about 20% of the participants reported being the victims of some form of bullying. This prevalence is found to be lower than the 25% found in a Taiwanese sample[15] or the 37.3% observed in a US sample[24], or the 23% found in another Korean sample[25]. These differences may reflect differences in the methods used to assess bullying, the clinical characteristics of the samples, or other characteristics such as gender or age. In a US study, bullying of students is defined as “when another student, or a group of students, say or do nasty or unpleasant things to the victim[24]. Although they described additional criteria of bullying, this broad definition might have a possibility for overestimation. We evaluated the being bullied experiences using self-administrated yes/no questionnaire according to participant’s subjective perception of bullying during the past 12 months, whereas other studies defined the bullying of victims by selecting several items of the standardized bullying questionnaires based on frequency such as ‘ often’ or’ two or three times a month’. Such simplicity and dependence of retrospective self assessment during relatively long period of our study might underestimate actual condition of bullying experiences. However, the prevalence revealed to be quite similar with another Korean study with middle school students [25].

Although it is common to classify bullying victims with respect to physical or verbal bullying [26–28], more than one type of bullying may be experienced by a victim, and the specific clustering of experiences of being bullied may depend on various factors associated with the victims or perpetrators. Zhang et al. reported that the correlation coefficient between the two bullying types was statistically significant, with adolescents who were the targets of one type of bullying being at higher risk of experiencing another type of bullying[27]. The present study clearly differentiated between two classes of bullying, with the presence of experiences of being physically bullied being the main determinant of this classification; however, verbal bullying was a common experience in both classes.

Several studies involving adolescents have investigated the association between various types of bullying in victims of suicide. One Dutch study reported that suicidal ideation was related with being bullied both directly and indirectly, with the associations being stronger for indirect than for direct bullying[5]. An Italian study investigated the effects of direct and relational victimization at school on suicidal ideation. The study showed that both direct and relational victimization at school were positively associated with suicidal thoughts, with relational victimization more strongly associated with suicide. A recent Taiwanese study classified bullying as either active (physical bullying, stealing belongings, etc.) or passive (social exclusion, name-calling, etc.), and found that both types were significantly associated with various kinds of mental health problems, including suicidal ideation and attempts [15]. Our classification of bullying shares some similarities with the classification of bullying as direct versus indirect; however, our classification is more realistic in that if was achieved by means of more case-based methodological approach with classification, and it therefore better reflects the complexities of real situations.

Cyber bullying is the newest form of bullying behavior and has expanded the temporal and spatial boundaries of bullying, allowing adolescents to be bullied 24 hours a day regardless of their location. A meta-analysis has suggested that cyber bullying is strongly related to suicidal ideation[29]. Surveys among US middle school students have found that cyber bullying is more strongly associated with depression[30] and suicidality[31] than is traditional bullying. Smith et al[32] reported that many cyber bullying victims were also victims of traditional bullying. Our bullying assessment included cyber bullying, and we found that it was common in both the physical and non-physical classes, though more prevalent in the non-physical than in the physical class. Our result suggests that cyber bullying is not a separate type of bullying, but is more associated with non-physical bullying that includes indirect or relational forms of bullying rather than physical bullying. We need to pay more attention to negative consequences including suicidal risk of cyber bullying.

School bullying is currently a serious social problem in Korea. Following the aggressive efforts of Korean society and the government to improve the monitoring and reporting of bullying, the overall rate of school bullying in Korea, particularly physical bullying, has decreased in recent years. Nevertheless, cyber and verbal bullying are harder for adults to detect, and there is objective evidence that these forms of bullying are increasing[33].Given the reality of this situation and the association of non-physical forms of bullying on suicidal behavior, there is an urgent need to protect students from bullying at school, an effort that must involve collaborations among schools, families and communities in carefully and comprehensively redefining the assessment of bullying. Schools and mental health professionals need to be sensitive to this “hidden” type of non-physical bullying and devote more attention to it.

We expected distinct types of bullying to exert distinct association on suicidal ideation and attempts among adolescents, but there were no significant differences in suicidal ideation and attempts between the two bullying classes in this cross-sectional study. In fact, we found no significant differences in victim characteristics between these classes, with the exception of gender. This suggests that there may be significant differences in characteristics of perpetrators. Aside from the dyadic relationship of victim and perpetrator, social and cultural factors may have an effect on co-occurring bullying experiences. Additionally, further longitudinal research with more detailed assessment methods may reveal significant differences between the two classes of bullying with respect to clinical outcomes for victims. A longitudinal study involving Swedish adolescents demonstrated that direct and indirect victimization predicted different profiles of psychological difficulties[16].

Studies conducted throughout the world have pointed to the dangers of bullying by emphasizing maladaptive outcomes for victims, including anxiety, loneliness, sadness, overcompliance, insecurity, and frustration [34–37]. In addition to these internalizing behaviors, the target of bullying may display externalizing problems such as impulsiveness and hyperactivity[38,39]. The present study showed significantly higher mean scores on the clinical subscales of the SDQ-Kr in both of the groups who experienced bullying relative to the group who did not, but we cannot determine whether this characteristic is a cause or a consequence of bullying.

The association between bullying and suicidality is receiving serious attention [13,17,40], as experiences of bullying result in more severe suicidal behaviors among adolescents [5,10,41]. As shown in our study, suicidal ideation was not related to either physical or non-physical bullying after adjustment was made for confounding effects. However, experiences with both bullying classes were strongly associated with suicide attempts. These results suggest that bullying is directly associated with more risky suicidal behaviors. Moreover, non-physical bullying had almost the same association with suicide attempts as physical bullying. This study supports the notion that being a victim of bullying is an important risk factor for attempted suicide regardless of the specific nature of those bullying experiences. A recent meta-analysis reported a significant positive association between suicidality and bullying victimization, with ORs of approximately 2–4[42]. The ORs for the association between suicide attempts and bullying victimization in this study were approximately 3, similar to previous results.

Although different classes of bullying showed similar risk factors for suicide attempts, our results showed significant clinical differences between two classes. A previous study has reported that different types of bullying were associated with different mental health problems[28]. These results suggest that different intervention strategies might be required for the two distinct classes of bullying and for the various bullying behaviors within each general type. Future research is required to gather data on the clinical characteristics and prognoses of the various types of bullying, with the ultimate aim of developing specific intervention strategies for victims suffering from psychiatric problems.

A further strength of the present study was the use of up-to-date statistical methodology methods in the analysis. The precise classification of bullying types and the determination of their association with suicidal behaviors enabled us to identify differences among bullying subgroups. LCA allowed us to identify the characteristics and complex patterns of each subgroup, which in turn may facilitate the development of more effective interventions targeted at specific groups. In psychiatry, LCA has been used to analyze symptom data from other neuropsychiatric disorders, most notably attention deficit–hyperactivity disorder [43–47] and obsessive–compulsive disorder [48,49]. Whereas a factor analysis looks for the underlying structure among specific variables, LCA can be used to find latent homogeneous groups of individuals, providing an additional dimension of analysis [48]. This has the advantage of potentially refining our understanding of adolescents’ experiences of being bullied and may improve our ability to identify the nature of bullying, provide specific targeted interventions, and predict outcomes. A large sample is required for LCA to provide a valid classification solution. Our study had a relatively large sample, which yielded explanatory power and the possibility of parsimony. Additionally, we conducted binomial logistic regression weighted by multi-group propensity scores to more precisely estimate the association of being bullied and to compare the effect size of the bullying types. The use of propensity scores is not yet a common practice in psychiatric research, though its application is increasing. Our data indicate that the use of propensity scores and related techniques may contribute to mental health studies with highly multi-dimensional compound datasets.

For careful interpretation of association between bullying victimization and suicidal behavior, we might consider bullying assessment method. A recent meta-analysis study suggests that bullying assessment method affects the association between bully-victim status and suicidal ideation [50]. When definitional and non-behavioral assessment methods were used, the largest effect size should have been shown [50]. In this study, we did not provide the accurate definition of bullying to the participants but assessed their experiences about being bullied through subjective perception of bullying by simple questions. We assume that the participants were well aware of the definition of bullying because every student should get regular bullying prevention education in the school and take part in the national survey for school bullying with providing accurate definition (e.q imbalance of power and repetition, specific types of bullying) of bullying twice a year in Korea. Although our assessment might reflect realistic perception of bullying among Korean adolescents, we cannot disregard the possibility of a significant error in estimation of actual victimization prevalence. A previous study reported that among students who self-reported being victims of bullying, approximately half were confirmed as actual victims. Student might misconstrue ordinary peer conflict as bullying [50]. Even the most widely adopted tool for bullying assessment, the Olweus Bully/Victimization Questionnaire, reported different responses with the California Bullying Victimization Scale that did not use the term “bullying,”, but included items that asked about its defining characteristics (repetition, intentionality, power imbalance)[51]. Therefore, simple assessment of bullying depending on self-report might be a major limitation of our study for proper interpretation. If we had assessed bullying with accurate definition and standardized measurement, larger effect size or different results might have been reported.

This study also has additional limitations. First, it used a clinical sample of individuals who had been referred to treatment due to psychological and behavioral issues and who were therefore a relatively high-risk group. Thus, our findings may not generalize to the present population of South Korea. Second, the prevalence of the classes identified by LCA may have been affected by our sample selection despite its size. Third, low reliability coefficient of conduct subscale was reported in the SDQ-Kr. Forth, the evaluation of suicidal behavior was self-administrated rather than based on clinical interviews or a standardized assessment. For assessing suicidal ideation, we only checked verbal expression of suicidal ideation, without including unexpressed suicidal ideation. There might be significant difference between talking about suicide and actual suicidal ideation. Finally, it is difficult to infer causal relationships from a cross-sectional study. More focused and longitudinal research would allow for more accurate identification of any causal association between peer bullying and suicidal behaviors.
